# [^18^F]DPA-714 PET imaging shows immunomodulatory effect of intravenous administration of bone marrow stromal cells after transient focal ischemia

**DOI:** 10.1186/s13550-018-0392-6

**Published:** 2018-05-02

**Authors:** Chengbo Tan, Songji Zhao, Kei Higashikawa, Zifeng Wang, Masahito Kawabori, Takeo Abumiya, Naoki Nakayama, Ken Kazumata, Naoyuki Ukon, Hironobu Yasui, Nagara Tamaki, Yuji Kuge, Hideo Shichinohe, Kiyohiro Houkin

**Affiliations:** 1 Department of Neurosurgery, Graduate School of Medicine, Hokkaido University, N15 W7, Kita-ku, Sapporo, 060-8638 Japan; 2 Advanced Clinical Research Center, Fukushima Global Medical Science Center, Fukushima Medical University, Fukushima, Japan; 3 Department of Tracer Kinetics and Bioanalysis, Graduate School of Medicine, Hokkaido University, Sapporo, Japan; 4 Department of Nuclear Medicine, Graduate School of Medicine, Hokkaido University, Sapporo, Japan; 5 Central Institute of Isotope Science, Hokkaido University, Sapporo, Japan; 6 Department of Integrated Molecular Imaging, Graduate School of Medicine, Hokkaido University, Sapporo, Japan; 7 Division of Clinical Research Administration, Hokkaido University Hospital, Sapporo, Japan

**Keywords:** Bone marrow stromal cell, Translocator protein, [^18^F]DPA-714 PET, Ischemic stroke, Inflammation

## Abstract

**Background:**

The potential application of bone marrow stromal cell (BMSC) therapy in stroke has been anticipated due to its immunomodulatory effects. Recently, positron emission tomography (PET) with [^18^F]DPA-714, a translocator protein (TSPO) ligand, has become available for use as a neural inflammatory indicator. We aimed to evaluate the effects of BMSC administration after transient middle cerebral artery occlusion (MCAO) using [^18^F]DPA-714 PET.

The BMSCs or vehicle were administered intravenously to rat MCAO models at 3 h after the insult. Neurological deficits, body weight, infarct volume, and histology were analyzed. [^18^F]DPA-714 PET was performed 3 and 10 days after MCAO.

**Results:**

Rats had severe neurological deficits and body weight loss after MCAO. Cell administration ameliorated these effects as well as the infarct volume. Although weight loss occurred in the spleen and thymus, cell administration suppressed it. In both vehicle and BMSC groups, [^18^F]DPA-714 PET showed a high standardized uptake value (SUV) around the ischemic area 3 days after MCAO. Although SUV was increased further 10 days after MCAO in both groups, the increase was inhibited in the BMSC group, significantly. Histological analysis showed that an inflammatory reaction occurred in the lymphoid organs and brain after MCAO, which was suppressed in the BMSC group.

**Conclusions:**

The present results suggest that BMSC therapy could be effective in ischemic stroke due to modulation of systemic inflammatory responses. The [^18^F]DPA-714 PET/CT system can accurately demonstrate brain inflammation and evaluate the BMSC therapeutic effect in an imaging context. It has great potential for clinical application.

## Background

In recent years, despite an improvement in the lifesaving rate of brain ischemic stroke, the numbers of patients whose sequelae of nervous symptoms are too severe for daily living are increasing [1]. With respect to the sequelae of brain infarction, the biggest issue is the difficulty associated with nerve regeneration after brain damage. On the other hand, brain damage-derived inflammation also plays an important role in the pathogenesis of ischemic stroke. It occurs immediately to induce an array of alterations in the immune response, including the infiltration of inflammatory cells from the peripheral circulation into the ischemic brain as well as the activation of resident inflammatory cells [2, 3]. Furthermore, recent clinical studies reported that stroke patients with a systemic inflammatory profile such as atherosclerosis, diabetes, and peripheral infection, can exhibit poorer outcomes [4, 5]. In addition, in many of stroke studies it remains indefinite about the effect of cerebral infarction on systemic immune organs and subsequent brain secondary damage. Finding an effective treatment for reducing stroke-associated injury and restoring neural function has become a burning problem that needs to be solved urgently. With this intention, the potential of cell-based therapy is being explored.

Bone marrow stromal cells (BMSCs), multipotent progenitors with the ability to differentiate into various types of cell lineages, have been considered to have potential application in ischemic stroke therapy. These cells can be easily harvested from the patient, cause no immunological reactions, and have a remarkable capacity for extensive expansion in vitro [6–9]. Recently, BMSCs have been shown to possess immunomodulatory effects to inhibit inflammatory processes [10–13]. We propose the following hypothesis: if ischemic injury occurs, especially following ischemia–reperfusion, pro-inflammatory cytokines produced by the activated microglia are released into the systemic circulation where they stimulate immune organs. These immune organs drive inflammation-mediated damage, thereby inducing more severe brain damage. The rapid use of BMSCs, with the stem cells distributing to lymphoid organs and the brain, could act to downregulate the hyper-inflammatory response and upregulate repair processes.

In recently years, a neural inflammatory biomarker, translocator protein (TSPO) [14–18], has been applied to positron emission tomography (PET). Due to the overexpression in activated microglia, TSPO is linked to inflammatory responses that occur after brain injury or neurodegenerative diseases. Evaluation of inflammatory condition by PET imaging maybe conducted to assess anti-inflammatory effect for cell therapy. Using a TSPO ligand [18F]DPA-714 PET/CT system in the present study, we aim to investigate the relationship between stroke and inflammation, and to clarify the immunomodulation ability of BMSCs in post-ischemic inflammation.

## Methods

### Preparation of rat BMSCs

BMSCs were isolated from 13-week-old F344/NSIc rats (Japan SLC, Inc., Shizuoka, Japan). Rats were initially anesthetized with 4% isoflurane in N_2_O/O_2_ (70:30) and were maintained via spontaneous ventilation with 2% isoflurane in N_2_O/O_2_ (70:30). The femora were aseptically dissected. Both ends were cut, the marrow was extracted and rinsed with 5 mL DMEM containing 10% fetal calf serum (FCS), 1% penicillin/streptomycin (P/S), and 10% heparin, using a 2.5 mL syringe [19]. The cell suspension was collected and centrifuged at 800×*g* for 5 min at 15 °C. The pellet was suspended in PBS, and subsequently, cells were seeded in a 175 cm^2^ flask with 25 ml DMEM containing 10% FCS and 1% P/S, and were cultured in an incubator with 5% CO_2_ at 37 °C. After 48 h, non-adherent cells were removed by changing the medium. The culture medium was replaced two or three times a week. BMSCs were passed three times for subsequent transplantation.

The BMSCs were lifted using 4 mL TrypLE Select (a recombinant trypsin substitute, Thermo Fisher Scientific, Inc., Waltham, MA, USA) and were incubated for 5 min. After fully agitating, cell suspensions were transferred into a test tube and centrifuged at 800×*g*, 5 min at 15 °C. The supernatant was decanted, and cells were gently resuspended using normal saline solution.

All animal experiments were approved by the Animal Studies Ethical Committee at Hokkaido University Graduate School of Medicine.

### Rat infarct model and intravenous cell administration

Male F344/NSIc rats (250–270 g) were used for the transient middle cerebral artery occlusion (tMCAO) model. Anesthesia was induced as described above. Rectal temperature was maintained at 37 °C throughout the surgical procedure using a temperature controller system (NS-TC10, Neuroscience, Inc., Tokyo, Japan).

During the operation, the right common carotid artery (CCA), the external carotid artery (ECA), and the internal carotid artery (ICA) were exposed. A silicon rubber-coated monofilament with a tip coating diameter of 0.37 mm (Doccol Corp., Redlands, CA, USA) was inserted into the right ECA and advanced into the ICA to block the middle cerebral artery (MCA) origin. After 90 min of MCAO, the suture was gently removed, and the ECA was coagulated to permit reperfusion [20, 21]. Cerebral blood flow (CBF) in the territory of the MCA was measured by Laser Doppler Flowmetry (OMEGAFLO FLO-C1; OMEGAWAVE, Tokyo, Japan) before and after MCAO [21]. At 90 min after reperfusion, the 18-point Neurological Severity Score (NSS) was assessed. Rats with a CBF reduction greater than 70% and with an NSS of more than nine points were included in this study.

An hour after reperfusion, 0.5 mL normal saline-diluted BMSC suspension (3 × 10^6^ cells) or normal saline was injected into the left saphenous vein using a 26 G syringe. Sham animals underwent the same surgical procedure without monofilament insertion. And only a normal saline injection (0.5 μl) was performed at one hour after operation.

### EdU staining

For cell labeling, 10 μM EdU (5-ethynyl-2′-deoxyuridine, Thermo Fisher Scientific, Inc., Waltham, MA, USA) was added to the culture medium 48 h before administration and the mixture was incubated with one part of the BMSCs. The labeled cells were trypsinized and resuspended in normal saline. One part of the EdU-BMSCs was used for smear examination, while the others were transplanted into rats. 12 h later, five rats were euthanized and their tissue was removed and sliced. The cells and tissue slices were stained using the EdU Alexa Fluor 555 Imaging Kit (Thermo Fisher Scientific, Inc.) in accordance with the manufacturer’s instructions. After this, slices were counterstained with Hoechst 33,342 (Thermo Fisher Scientific, Inc.).

### Neurological and body weight assessments

A functional assessment battery was performed at 3 h, 1, 3, 5, 7, 9, 11, and 13 days after MCAO to give the NSS (BMSC group, *n* = 10; vehicle group, *n* = 10). Briefly, the 18-point scale score system comprises four different domains: (1) motor, (2) sensory, (3) reflex, and (4) balance tests [22]. In the series of assessments, one score point is awarded for an inability to perform tasks or for the lack of a tested reflex. Thus, a higher score represents a more severe nerve injury [22]. Animal body weight was also assessed at 1, 3, 5, 7, 9, 11, and 13 days after surgery (sham surgery group, *n* = 3; BMSC group, *n* = 10; vehicle group, *n* = 10).

### Quantitative analysis of ischemic volume and lymphoid organ weight

Rat brains were harvested for 2,3,5-triphenyltetrazolium chloride (TTC) staining analysis to evaluate infarction volume at 14 days after MCAO (BMSC group, *n* = 15; vehicle group, *n* = 15). Briefly, six 2 mm-thick serial coronal sections were cut and stained with 2% TTC (Sigma-Aldrich Chemie GmbH, Buchs, Switzerland) at 37 °C for 15 min. Each section was scanned using a high-resolution scanner (Epson, GT-X820, Nagano, Japan) and was quantified by ImageJ (NIH, Bethesda, MD, USA). Infarction volumes were calculated as the percentage volume of the left normal hemisphere, according to the following formula: (left hemisphere volume − right non-infarct volume)/left hemisphere volume (%) [21, 23]. Rat spleens and thymuses were also obtained for analysis of weight at 14 days after MCAO (sham surgery group, *n* = 4; BMSC group, *n* = 15; vehicle group, *n* = 15). Moreover, 11 extra rats were added to the vehicle group. They had shorter occlusion time (60 min) to obtain small brain infarction volume (from 9 to 30%) than the normal MCAO (90 min) rats.

### PET/CT scans and autoradiography imaging

[^18^F]DPA-714 PET/CT imaging was carried out for each animal (BMSC group, *n* = 4; vehicle group, *n* = 3) at 3 and 10 days after MCAO. The PET/CT images were obtained using the Inveon small animal imaging system (Siemens Medical Solutions, Knoxville, TN, USA). The PET component consisted of 1.5 × 1.5 × 10 mm lutetium oxyorthosilicate crystal elements with a ring diameter of 16.1 cm, providing a 10 cm transaxial and a 12.7 cm axial field of view [24]. Rats anesthetized with isoflurane were injected with 13.2 ± 0.8 MBq [^18^F]DPA-714 via the tail vein. They were then returned to their cages and allowed to move freely for 30 min. Rats were subsequently anesthetized with isoflurane and scanned by the PET/CT system for 20 min. The PET datasets were reconstructed as reported previously [25]. Region of interest (ROI) analysis was conducted in the infarction area, and the control was symmetrically placed in the contralateral hemisphere. And then, counting rates were converted to standardized uptake values (SUVs). The ratio of ipsilateral to contralateral radioactivity was calculated, using IDL (Research Systems, CO, USA) and ASIPro VM (Concorde Microsystems, Knoxville, TN, USA). In the autoradiography (ARG) experiment, rats were euthanized at 90 min after [^18^F]DPA-714 injection. The brains were quickly removed and cut into six 2-mm-thick coronal slices. The second and fourth slices were exposed to a phosphor imaging plate (Fuji Photo Film Co., Ltd., Tokyo, Japan) together with a set of calibrated standards. After the exposure, the imaging plate was scanned with FLA 7000 BioImaging Analyzer (Fujifilm Life Science, Stamford, CT, USA) and images were analyzed using Multi Gauge V3.2 (Fujifilm Life Science).

### Immunohistochemistry and apoptosis assay

Animal brains, spleens, thymuses, and lymph nodes were obtained for histological analysis. In the BMSC group, 20 rats were euthanized at 1 day (*n* = 5), 3 days (n = 5), 7 days (n = 5), or 14 days (n = 5) after cell administration in addition to subjects in the vehicle group. Animals that had a sham operation (*n* = 3) were euthanized at 14 days after MCAO.

Immunohistochemistry was performed as previously described [19]. Tissue was removed and stored in 4% paraformaldehyde for two days and embedded in paraffin Coronal sections that were 4 μm thick were prepared for subsequent analysis. Each section was treated with a primary antibody against Iba1 (rabbit polyclonal antibody, 1:1500 dilution, Wako, Osaka, Japan) or CD8α (mouse monoclonal antibody, 1:150 dilution, EXBIO, Prague, Czech Republic) at 4 °C overnight, and was then incubated with Alexa Fluor 594 goat anti-rabbit antibody or Alexa Fluor 488 goat anti-mouse antibody (1:200 dilution, Thermo Fisher Scientific, Inc.) at room temperature for 1 h. Brain sections were co-incubated with a mouse monoclonal anti-CD68 antibody (1:200, Abcam, Cambridge, UK) and an anti-Iba1 antibody at 4 °C overnight. On the following day, sections were incubated with Alexa Fluor 488 and Alexa Fluor 594. Images were obtained using a fluorescence microscope (BZ-X700, KEYENCE, Osaka, Japan).

Sections of the spleen and lymph nodes were dyed using the TUNEL Alexa Fluor 647 assay kit (Thermo Fisher Scientific, Inc.) in accordance with the manufacturer’s instructions. Sections were subsequently stained using an anti-CD8α antibody at room temperature for 1 h and were labeled with Alexa Fluor 488.

### Statistical analysis

All data are expressed as the mean ± SD. Results of NSS, body weight, and brain infarction volume were compared using unpaired *t* tests (Fig. 2a–c). Results of spleen weight and thymus weight were compared using one-factor analysis of variance (ANOVA) followed by Bonferroni-Dunn analysis (Fig. 3b, c). Results of SUV were compared using Kruskal-Wallis analysis (Fig. 4b, c). Linear regression was performed to evaluate the correlation between brain infarction volume and lymphoid organ weight. Significance was assumed at *P* < 0.05.

## Results

### Distribution of BMSCs in organs

**Fig. 1:**
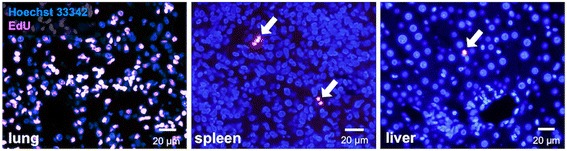
Organ distribution of BMSCs stained by EdU. Photomicrographs show the migrating cells in lung (left), spleen (center), and liver (right) at 12 h after MCAO, respectively. Pink, EdU and blue, Hoechst 33,342. Scale bars, 20 μm

Following EdU staining, fluorescence micrographs demonstrated that approximately 60% of BMSCs were labeled. At 12 h after cell administration, a large quantity of EdU-BMSCs was widely distributed in the lung, and some cells had arrived in the spleen and liver (Fig. 1). However, no cell was found in the brain parenchyma throughout the observation period.

### BMSCs improve neurological symptoms and body weight loss

**Fig. 2:**
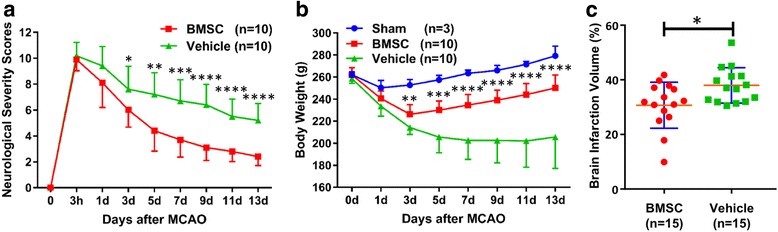
Therapeutic effects of BMSCs. Panels **a**–**c** show the neurological severity score (**a**), body weight (**b**), and the brain infarction volume (**c**). Sham group, blue; BMSC group, red; and vehicle group, green. *P < 0.05, **P < 0.01, ***P < 0.001, and ****P < 0.0001; BMSC vs. vehicle

The NSS demonstrated severe motor deficits at 3 h after MCAO in both groups (BMSC group, 9.90 ± 0.88; vehicle group, 10.20 ± 1.03). Afterward, although animals in the vehicle group showed an improvement in motor deficits, they had distinct paralysis symptoms at 13 days after MCAO (5.20 ± 1.32). Animals in the BMSC group, on the other hand, showed significant improvement in the NSS compared to subjects in the vehicle group from 3 days after MCAO, and they had almost completely recovered at 13 days (2.40 ± 0.70, Fig. 2a).

After MCAO, every rat demonstrated body weight loss. The animals in the sham group also had weight loss at 1 day after surgery. In the vehicle group, each animal had severe weight loss and maintained a low weight, with some rats losing more than 30% of their body weight. In the BMSC group, however, body weight began to recover and showed significant improvement compared to the vehicle group from 3 days after MCAO (Fig. 2b).

### BMSCs reduce ischemic lesion volume

Stroke-induced brain lesions were confirmed by TTC staining at 14 days after MCAO. Cerebral infarction was widely distributed in the ipsilateral cerebral cortex and striatum in both groups. The mean infarction volume in vehicle-treated rats was 38.0 ± 6.5%. In contrast, the infarction volume was significantly reduced at 30.7 ± 8.4% in the BMSC group (*P* = 0.0132, Fig. 2c).

### BMSCs rescue stroke-induced alterations in lymphoid organ weight

**Fig. 3:**
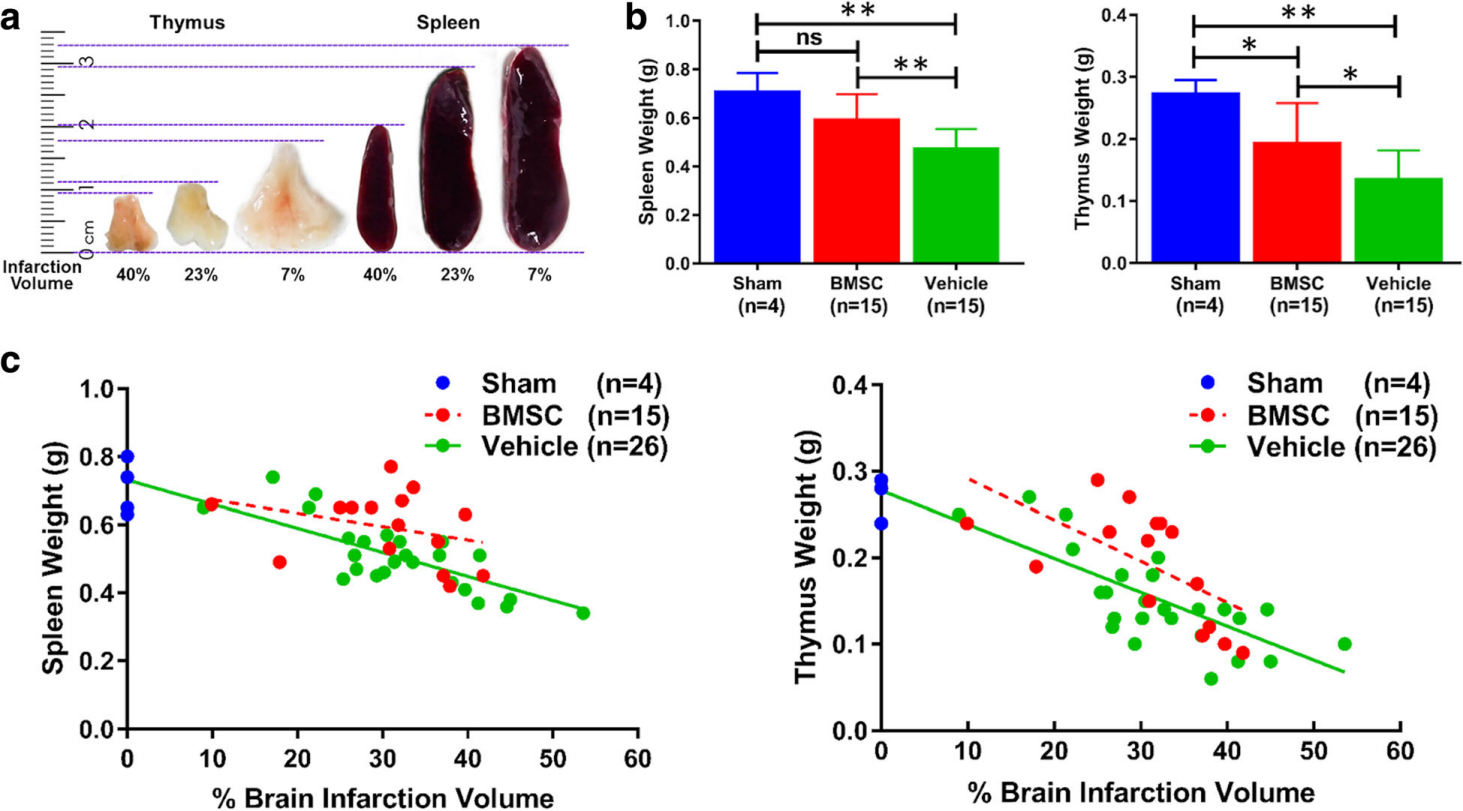
Relationship between lymphoid organ size and brain infarction volume. Panel **a** shows representative photographs of rat lymphoid organs after MCAO. Panel **b** shows the effects of MCAO and BMSC administration on the organ weight (left, spleen; and right, thymus). Panel **c** shows the correlation between infarction volume and organ weight (left, spleen; and right, thymus). Sham group, blue; BMSC group, red; and vehicle group, green. *P < 0.05, **P < 0.01, and ns, not significant

It was apparent that the loss in size of the spleen and thymus positively correlated with brain infarction volume (Fig. 3a). Compared with the sham group, animals in the vehicle group had lost two-thirds of their spleen weight and half of their thymus weight at 14 days after MCAO, which constituted a significant change (*P* < 0.01 in both groups). However, BMSC treatment significantly suppressed this organ weight loss by approximately 50% (spleen, *P* < 0.01 and thymus, *P* < 0.05). In addition, no statistical differences in spleen weight were observed between the sham and BMSC groups (Fig. 3b).

With respect to the correlation between lymphoid organs and the brain, infarction volume was negatively correlated with spleen and thymus weight in sham and vehicle groups (spleen, *r* = − 0.8365, *r*^2^ = 0.6997; thymus, *r* = − 0.8319, *r*^2^ = 0.692). In the BMSC group, although the slopes of the linear regression equations did not differ significantly from sham and vehicle groups, the intercepts were significantly different (spleen, *P* = 0.0046 and thymus, *P* = 0.0120; Fig. 3c).

### PET imaging reveals immunomodulatory effects after BMSC administration

**Fig. 4:**
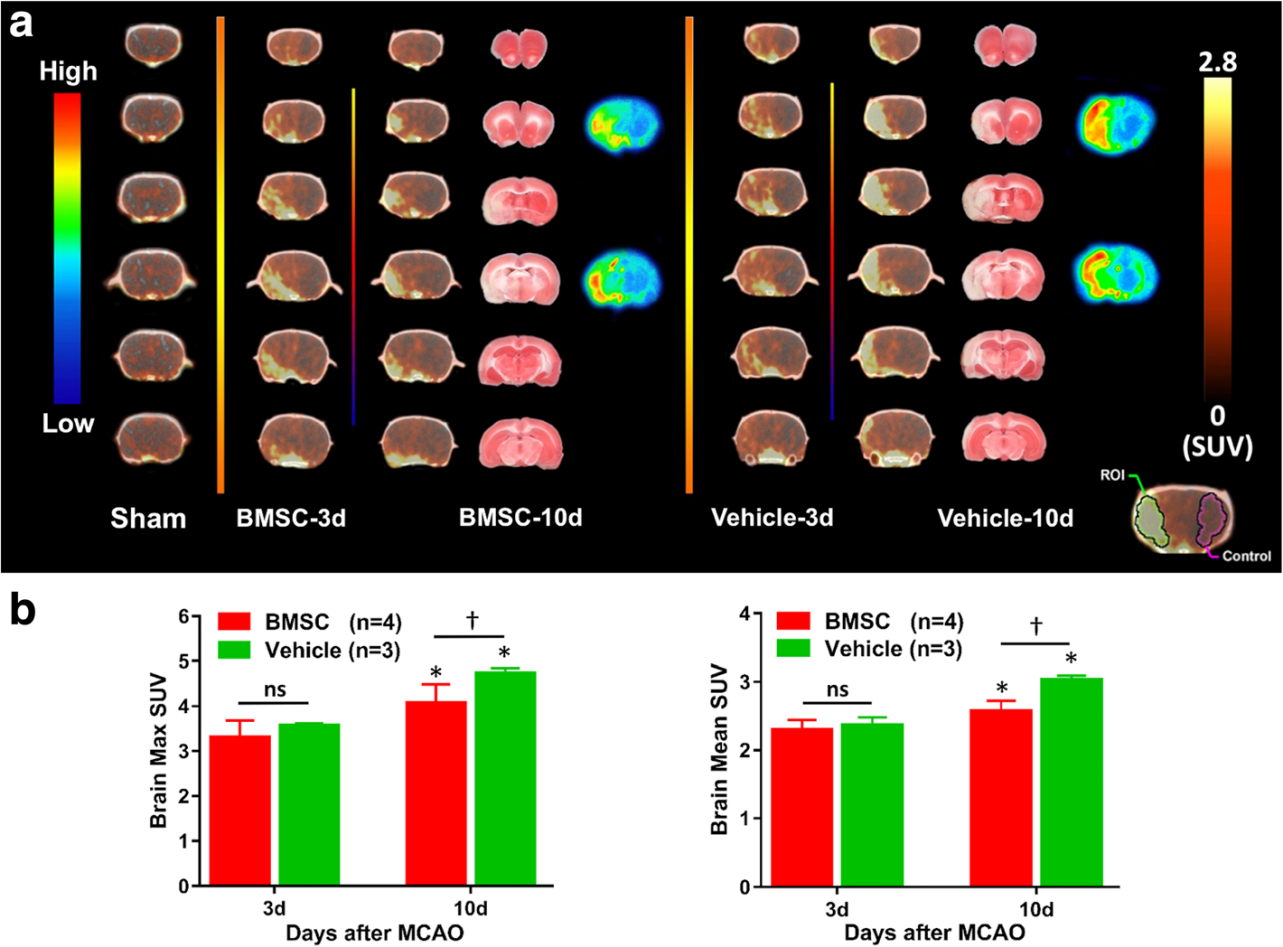
[^18^F]DPA-714 PET images. Panel **a** shows representative PET images (yellow and brown), TTC staining (red and white), and ARG (rainbow colors) of rat brains. Color bars indicate the scale of SUV in PET images (right) and the qualitative scale in ARG (left). Panel **b** shows the max SUV (right) and the mean SUV (left). 3d and 10d indicate the day after MCAO. BMSC group, red; and vehicle group, green. *P < 0.05, 3d vs. 10d; †*P* < 0.05 and ns, not significant, BMSC vs. vehicle

[^18^F]DPA-714 PET showed that inflammatory changes occurred around the ischemic lesion 3 days after MCAO in both vehicle and BMSC groups (Fig. 4a). However, no statistical differences were observed between the groups regarding the maximum (max) SUV and the mean SUV (BMSC group, max SUV = 3.30 ± 0.38, mean SUV = 2.30 ± 0.14; vehicle group, max SUV = 3.57 ± 0.06, mean SUV = 2.37 ± 0.12, Fig. 4b). At 10 days after MCAO, the SUV was significantly increased in the vehicle group (max SUV, 4.73 ± 0.16, *P* = 0.04311; mean SUV, 3.03 ± 0.06, *P* = 0.04311). Although the max SUV and mean SUV were also increased in the BMSC group at 10 days (max SUV, 4.08 ± 0.41, *P* = 0.03839; mean SUV, 2.58 ± 0.15, *P* = 0.03719, respectively), BMSC administration inhibited inflammatory progression and expansion, compared to the vehicle group (max SUV, *P* = 0.04768; mean SUV, *P* = 0.03075). These findings corresponded with TTC and ARG images (Fig. 4a). On the other hand, no significant differences in PET images of body were observed among sham, vehicle-3d, and vehicle-10d rats (data not shown).

### BMSCs inhibit stroke-induced histological alterations

**Fig. 5:**
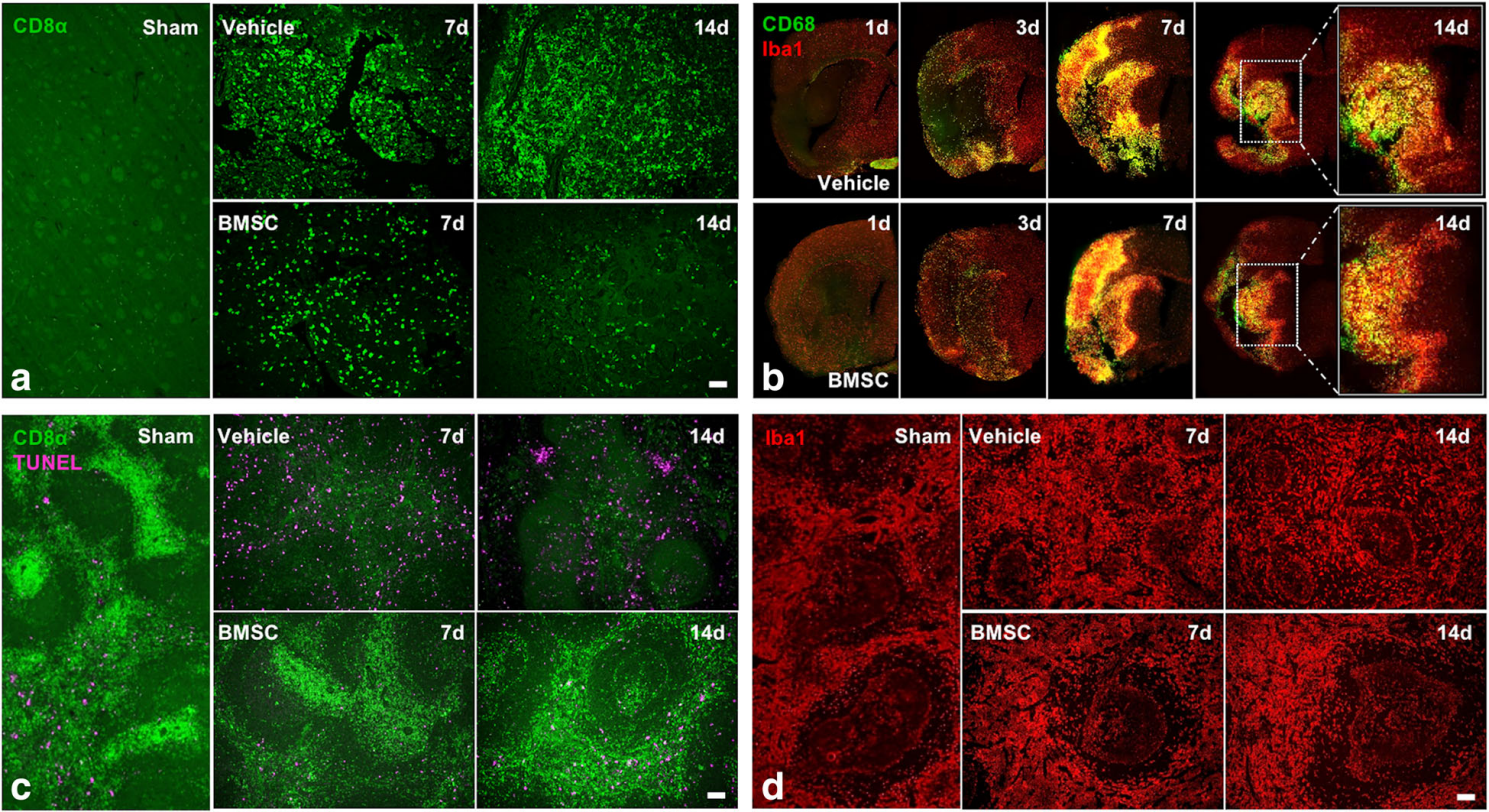
Histology of the brain and spleen. Panels **a** and **b** show photomicrographs of CD8α^+^ cells (green in **a**), CD68^+^, and Iba1^+^ cells (green, CD68; red, Iba1 in **b**) in the brain. Panels **c** and **d** show photomicrographs of CD8α^+^ and apoptosis cells (green, CD8α; pink, TUNEL in **c**) and Iba1^+^ cells (red in **d**) in the spleen. 1d, 3d, 7d, and 14d indicate the day after MCAO. Scale bars, 100 μm

Immunohistochemical analysis showed the appearance of and gradual increase in CD8α^+^ T cells in cerebral infarction after MCAO (Fig. 5a). With aggravation of the injury, the number of CD8α^+^ T cells peaked at 14 days after MCAO. In the BMSC group, on the other hand, fewer CD8α^+^ T cells were seen compared to the vehicle group at 14 days after MCAO (Fig. 5a).

On the first day after insult, double immunostaining for Iba1 (a pan-microglia marker) and CD68 (a marker for the activated inflammatory phenotype) showed the presence of a small number of microglia around the infarction area in both vehicle and BMSC groups (Fig. 5b). Subsequently, double-positive cells for Iba1 and CD68, which was known as activated microglia, were gradually distributed, increased in number, and were maintained at a high level throughout the entire lesion. At 14 days after insult, tissue cavitation appeared around the infarction area which was surrounded by a large amount of CD68^+^ activated microglia in the vehicle group. By contrast, there were fewer microglia expressing the CD68^+^ phenotype in the outer portion of the infarction area in the BMSC group (Fig. 5b).

**Fig. 6:**
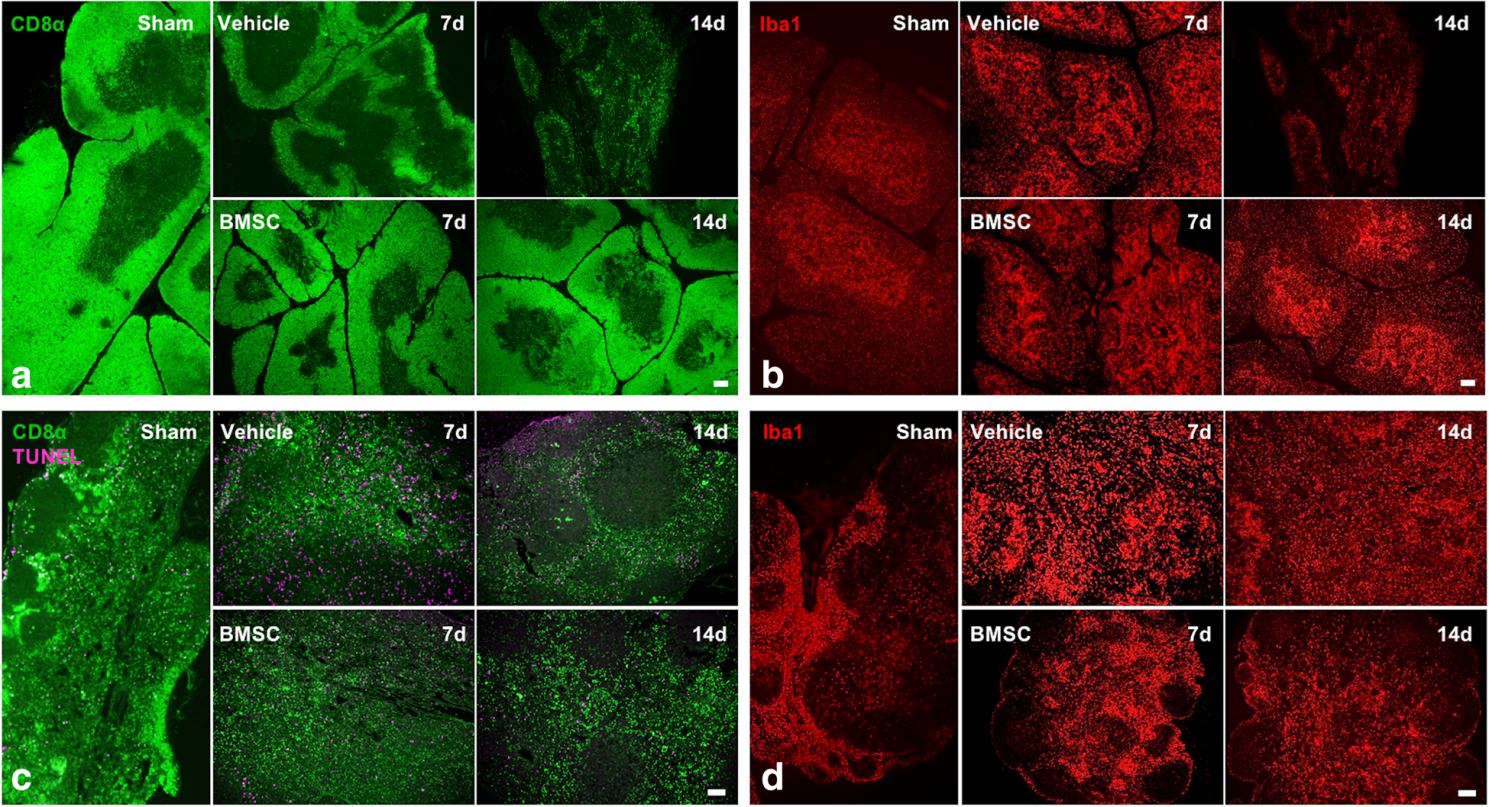
Histology of the thymus and lymph nodes. Panels **a** and **b** show photomicrographs of CD8α^+^ cells (green in **a**) and Iba1^+^ cells (red in **b**) in the thymus. Panels **c** and **d** show photomicrographs of CD8α^+^ and apoptosis cells (green, CD8α; pink, TUNEL in **c**) and Iba1^+^ cells (red in **d**) in lymph nodes. 7d and 14d indicate the day after MCAO. Scale bars, 200 μm (**a** and **b**) and 100 μm (**c** and **d**)

In the white pulp of the spleen (Fig. 5c), the cortex of the thymus (Fig. 6a) and the deep cortex of the lymph nodes (Fig. 6c), the number of CD8α^+^ T cells markedly decreased after MCAO in the vehicle group. TUNEL staining showed that the number of apoptotic cells increased in the spleen and lymph nodes following MCAO. As the number of CD8α^+^ T cells decreased, tissue atrophy began in the main residence area of the cells. In their place, Iba1^+^ macrophages acted as the main cell type in the spleen (Fig. 5d), thymus (Fig. 6b), and lymph nodes (Fig. 6d). The percentage of macrophages declined at 14 days after MCAO in the thymus only (Fig. 6b). In the BMSC group, these immune changes after cerebral ischemia, that is, a decrease in CD8α^+^ cells, an increase in TUNEL^+^ cells, tissue atrophy in the lymphoid organs, and a decrease in Iba1^+^ macrophages at 14 days in the thymus, were dramatically suppressed (Figs. 5c, d and 6).

## Discussion

Inflammation is a hallmark of the pathogenesis of ischemic stroke [26, 27]. Previous studies have indicated that ischemic stroke can induce rapid activation of microglia, resulting in an acute and prolonged inflammatory process that characterizes the brain response [28, 29]. It is known that infarct deterioration depends on microglia-mediated cytokines released around the peri-infarct area in the early phase [30]. These cytokines can trigger an inflammatory cascade reaction that aggravates brain edema and nerve injury, leading to necrosis of ischemic tissue.

In the present study, we found a negative correlation between infarction volume and lymphoid organ size. Regarding the atrophy of lymphoid organs, histological assessment showed that the number of CD8α^+^ cytotoxic T cells rapidly declined in their cell-rich region and the number of apoptotic cells increased in the spleen and lymph nodes after cerebral ischemia. Meanwhile, a large quantity of infiltrating cells appeared around the brain infarction area. CD8α^+^ T cells could be released into the circulation, which could, in turn, contribute to the secondary inflammatory cascade in the brain to induce more severe damage. We consider the correlation between infarction volume and lymphoid organ size an indication of the existence of a vicious cycle in vehicle and sham groups. Thus, a larger infarct size leads to more pronounced atrophy of lymphoid organs, and greater numbers of released CD8α^+^ T cells induce more severe secondary brain damage.

Recently, it has been noted that BMSCs can regulate inflammation by direct cell–cell contact and secretion of anti-inflammatory factors via a series of mechanisms, including both the innate and adaptive immune responses [31, 32]. In the present study, there was a significant improvement in neurological function and in body weight after cell administration. Histological analysis revealed that the BMSCs had migrated into the lungs, spleen, and liver at 12 h after cell transplantation. The process underlying release of pro-inflammatory cells and tissue atrophy was subsequently suppressed in lymphoid organs. Previous studies also confirmed that BMSCs could enter the body organs within a short time period after administration and took 10 days to reach peak levels in the brain [2]. Furthermore, in a study conducted by Yang et al. where multipotent adult progenitor cells (MAPCs), bone marrow-derived stem cells, were used in stroke models, large numbers of these cells had migrated to the lungs, spleen, and liver at day 1 after injection. By day 3, the number of cells began to decrease in these organs. However, no cells were observed in the brain [33]. They advocated that immunomodulation of the splenic response by the intravenous administration of the cells might create a more favorable environment for brain repair after stroke. In addition, BMSCs are associated with reduced numbers of CD8^+^ T cells and correspondingly increased numbers of apoptotic T cells in peripheral blood [32, 34]. Moreover, administration of BMSCs can also promote macrophage polarization, inducing a change from the activated inflammatory phenotype (M1) to the anti-inflammatory phenotype (M2) [35]. In the present study, we confirmed that inflammatory CD68^+^ microglia, which indicate the M1 phenotype, was the major phenotype around the injured area in the vehicle group. Conversely, in the BMSC group, there were more microglia without the CD68^+^ phenotype in the outer portion of the infarction area at 14 days after MCAO. This suggests that many microglia may have undergone a change in polarization to the M2 phenotype, thereby reducing secondary brain damage, as brought about by the immunomodulatory effect on the systemic inflammatory response. Furthermore, the histological findings in the brain correspond with the [^18^F]DPA-714 PET imaging data. Ory et al. reported that CD68^+^ microglia showed the immunoreactivity against TSPO, for which [^18^F]DPA-714 was a specific ligand, in the brain of a rat local neuroinflammation model [36].

The findings of the [^18^F]DPA-714 PET imaging showing that the inflammatory level in or around the brain damage was reduced in the BMSC group have two possible interpretations. Firstly, the PET findings could be the effect of the reduction in infarct size. Secondly, the decreased inflammatory level brought about the reduction in infarct size. We can consider both of these to be correct. Thus, we suggest that the immunomodulatory effect seen after cell administration could break a vicious cycle between ischemic damage and the inflammatory response. Evidence of this is the correlation between infarction volume and lymphoid organ size (Fig. 3d, e). If any therapeutic effect apart from immunomodulation, for example, an antioxidant effect, could affect the brain damage, the regression line would be identical to the one in the vehicle/sham groups. However, the line was shifted upward here. We, therefore, believe that the regression line in the vehicle/sham group is an expression of the previously mentioned vicious cycle, with the shift of the line evidence of its disruption or immunomodulation. Thus, our present results suggest that a strong systemic immunomodulatory effect arises following cell administration, possibly resulting in a local immunomodulatory effect in the brain and concomitant rescue of secondary ischemic damage.

It is known that TSPO is mainly located on the outer mitochondrial membrane [37]. Recently, there has been a focus on its role in immunomodulation, steroid synthesis, and apoptosis [37, 38]. Under normal physiological conditions, TSPO is maintained at a low level in the central nervous system. However, it is highly expressed in activated microglia in response to brain injury and inflammation [17, 14]. A previous study reported that TSPO was also expressed in the heart, kidneys, lungs, and spleen [39, 40]. [^18^F]DPA-714, a specific ligand for TSPO, is currently receiving attention as a potential neuroinflammatory indicator for PET imaging. Our present results using [^18^F]DPA-714 PET imaging show that TSPO is increased in the ischemic area. Moreover, this imaging revealed that BMSC administration inhibited inflammatory progression. This finding suggests that BMSC therapy could have anti-inflammatory potential against ischemic stroke. Thus, [^18^F]DPA-714 PET may be a promising modality to evaluate the inflammatory level of brain damage and assess the therapeutic benefits of cell therapy in ischemic stroke. Moreover, we considered that the modality would also be promising for the evaluation of cell-based therapy against other disorders. For examples, Bernards et al. reported that [18F]DPA-714 was suitable for studying inflammation in inflammatory bowel disease models [41]. Thus, our results suggested that [18F]DPA-714 might be applicable to clarify the immunomodulation ability in cell-based therapy against various inflammatory disorders.

In the present study, the number of animals included in the PET analysis (*n* = 4 for BMSC and *n* = 3 for vehicle) is small and it is the limitation of the study. However, we think that the number of animals is enough to evaluate the difference between two groups, because we obtained the PET findings at two time points in each animal. If it were histological analysis, we might need a couple of dozen animals to obtain the same conclusion. Moreover, our previous articles showed the solidity of the data which were obtained using the small-animal PET/SPECT/CT system [25] and the utility of the PET/SPECT analysis in the pre-clinical study for the cell therapy against stroke [24, 42]. The advantages of PET analysis are obvious.

## Conclusions

In summary, we demonstrated that brain infarction not only affects the brain, but also causes systemic inflammatory responses to aggravate neurological deficits after stroke. BMSC transplantation can inhibit brain inflammation and suppress lymphoid organ activation. The [^18^F]DPA-714 PET/CT system can accurately demonstrate brain inflammation and evaluate the BMSC therapeutic effect in an imaging context. This may be a good modality for clinical applications.
